# Engineering Elite Swimming Start Performance: Key Kinetic and Kinematic Variables with Reference Values

**DOI:** 10.3390/bioengineering13020180

**Published:** 2026-02-03

**Authors:** Dennis-Peter Born, Lina Nussbaumer, Markus Buck, Jesús J. Ruiz-Navarro, Michael Romann

**Affiliations:** 1 Swiss Development Hub for Strength and Conditioning in Swimming, Swiss Aquatics—National Swimming Federation, 3048 Worblaufen, Switzerland; lina.nussbaumer@swiss-aquatics.ch (L.N.); markus.buck@swiss-aquatics.ch (M.B.); 2 Department for Elite Sport, Swiss Federal Institute of Sport Magglingen, 2532 Magglingen, Switzerland; 3 Faculty of Science and Medicine, University of Fribourg, 1700 Fribourg, Switzerland; 4 Human Performance and Sports Science Laboratory, Faculty of Sport Sciences, University of Murcia, 30720 Murcia, Spain; 5 Aquatics Lab, Department of Physical Education and Sports, Faculty of Sport Sciences, University of Granada, 18071 Granada, Spain

**Keywords:** adolescents, benchmarks, competitive swimming, junior, normative values, PCA, talent

## Abstract

To provide deeper insights into the complex and multidimensional nature of swimming start performance, the present study aimed to determine its key performance indicators (KPIs) and provide percentile-based reference values for elite junior and adult swimmers. Hence, routine performance analysis data of Swiss junior and senior national team members were analyzed, including multiple European champions, World champions, Olympic medalists and a World record holder (n = 136, age: 18.3 ± 3.6 [13–32] years, World Aquatics swimming points: 761 ± 73 [609–1061]). All kinetic and kinematic variables measured by the instrumented starting block were analyzed, and variables with pairwise correlation > 0.80 were clustered using principal component analysis with orthogonal Varimax rotation, retaining components with Eigenvalue > 1.0 and factor loadings > 0.6. The highest loaded variables of each component were used as independent variables, alongside the variables with low co-variance, to determine KPIs with multiple linear regression analysis. As such, peak and average power (*p* ≤ 0.05), front horizontal and total vertical peak forces (*p* ≤ 0.04), timing of peak power and rear horizontal forces (*p* ≤ 0.02), resultant grab forces and their timing (*p* ≤ 0.05), center-of-gravity height at take-off (*p* = 0.03), take-off horizontal and vertical velocity (*p* = 0.02), resultant entry velocity (*p* = 0.01), entry time (*p* < 0.01), distance before the first kick (*p* < 0.01), maximal swimming depth (*p* = 0.02) and distance before breaking through the water surface (*p* < 0.01) showed a significant effect on the dependent variables (15 m start time). In conclusion, swimmers should maximize power and force production peaking earlier and grab forces peaking later during the block phase. They should increase take-off and entry velocities, distance before the first undulating kick, maximal swimming depth and underwater distance.

## 1. Introduction

Competition performance is a complex construct of multiple physiological and biomechanical factors that interact with each other [1,2,3]. Coaches use modern technologies to improve their understanding of these components and their interaction [2,4,5]. In swimming, motion analysis is particularly important as crucial movements occur at high velocities (>4 m/s) during block and flight phases or under the water surface, and are therefore difficult to assess with the naked eye [6]. The resulting kinetic and kinematic variables shed light on the complexity of start performance and reveal the various components, i.e., block-, flight-, entry-, underwater-phase, emersion and transition to full-stroke swimming [7], and their contribution to fast 15 m start times, a crucial success factor in competition [8,9].

Previous studies have revealed interesting insights into start performance using self-build or commercially available starting blocks with incorporated force plates, together with over- and underwater camera systems [7,10,11,12]. These sophisticated methodologies provide a comprehensive overview of the movement, with kinetic variables obtained during the block phase and kinematic variables calculated both over- and underwater. However, the volume of information provided by the large number of kinetic and kinematic variables is overwhelming when using those methodologies during the practice of competitive swimmers. To facilitate the translation of kinetic and kinematic motion analysis into practical feedback, it is necessary to determine the most relevant variables [13].

Therefore, recent studies used principal component analysis (PCA) to reduce the dimensions and cluster the data [7]; however, they included data from freestyle (FR) and butterfly (BU) swimmers only [7]. Others successfully determined the kinetic and kinematic key performance indicators (KPIs) with PCA in a sample of high-level swimmers; however, there was a reduced generalization of the results due to low sample size (n = 15) [11]. Although Peterson Silveira and co-workers [12] focused on breaststroke swimmers, their analysis of kinetic and kinematic parameters was limited to the 5 m mark and also based on low sample size (n = 13). Therefore, to translate the kinetic and kinematic data into practice and facilitate the interpretation of performance analysis data, a comprehensive analysis and determination of KPIs would include all four swimming strokes, a large sample size of high-level swimmers and reference values across a representative range of junior and adult national team members.

Therefore, the aims of the present study were to (1) identify the KPIs associated with faster swimming start performance in a sample of top-elite swimmers (national team members), (2) apply PCA as a dimension reduction technique to determine the most relevant kinetic and kinematic variables across all four swimming strokes, and (3) provide percentiles as references values to support the evaluation and interpretation of performance analysis data specific to sex. The hypothesis was that PCA would effectively reduce data dimensionality and cluster related variables, thereby enabling the identification of the most relevant kinetic and kinematic KPIs associated with faster swimming start performance, as well as providing practical interpretation guidelines for performance analysis data.

## 2. Materials and Methods

### 2.1. Study Design

The present study followed a structured and data-driven approach to identify KPIs of swimming start performance in a group of top-elite swimmers. All kinetic and kinematic variables provided by an instrumented starting block named Kistler performance analysis system for swimming (KiSwim, Kistler, Winterthur, Switzerland) were included. Dimensionality was then reduced with PCA by summarizing correlated variables into a smaller number of interpretable components. Variables with high loadings on these components were subsequently entered into multiple linear regression analysis, with a 15 m start time as dependent variable. The sequential analytical approach allowed identification of KPIs while accounting for multicollinearity, minimizing redundancy and maintaining practical interpretability for applied performance analysis.

### 2.2. Subjects

A total of 136 swimmers, all of them members of the Swiss junior and adult national swimming teams, which include multiple European champions, World champions, Olympic medalists and a World record holder, participated in the present study (females n = 67, males n = 69, age: 18.3 ± 3.6 [range: 13–32, median: 17.0] years, World Aquatics swimming points: 761 ± 73 [range: 609–1061, median: 761]). All subjects involved in the study—their legal guardians in case of minor-aged athletes—provided informed consent for the use of their performance data in scientific analyses. The study was conducted according to the code of conduct of the World Medical Association for medical studies involving human subjects (Declaration of Helsinki) and was pre-approved by the institutional review board of the Swiss Federal Institute of Sport Magglingen (registration number: 199-LSP-100523).

### 2.3. Data Collection

The study analyzed start performance data that were collected with the KiSwim (Table 1) at the Swiss national team’s routine performance analyses at the beginning of each macro cycle. Before each test session, the swimmers completed their standardized dry-land preparation, followed by 15 min of in-water warm-up, including aerobic swimming, technical drills and short sprints.

During each test session, swimmers performed two start trials from the KiSwim starting block using their main swimming stroke. The trial with the fastest 15 m time was used for the analysis to reduce random error, minimize the impact of technical mistakes and provide a robust measure of start performance. The two trials were separated by a 4–5 min rest period based on the standard elite swimmers’ training protocols. While invasive fatigue monitoring (e.g., blood lactate concentration) was not conducted, the fastest trial typically occurred randomly in either the first or second attempt, indicating that recovery between trials was sufficient. Also, Tor et al. [14] reported excellent reliability (ICCs > 0.90) for swimming start performance with only two minutes of rest between trials, demonstrating that top-elite swimmers recover rapidly and that trial order does not systematically influence performance. The force plates, which are integrated into the KiSwim starting block, measured kinetic data of the front and rear leg separately, grab forces, and wall reaction forces during backstroke starts at 500 Hz (Type 9027C/9028C, Kistler, Winterthur, Switzerland). The force plates were synchronized to the starting signal (Infinity Start System; Colorado Time Systems, Loveland, CO, USA), as well as one overwater (0.95 m above) and four underwater (0.95 m below the water surface) cameras (Prosilica GC660C; Allied Vision Technologies, Stadtroda, Germany). The cameras were located perpendicular to the swimming lane at the 1.5 m, 5 m, 10 m and 15 m marks and collected video footage at 100 fps. The calibration procedure of the KiSwim [11] and semiautomated postprocessing with the customary software (version 10.0, Kistler Performance Analysis System—Swimming Starts and Turns) has been described in detail before [15].

### 2.4. Statistical Analysis

The statistical analyses were conducted with the JASP (version 0.19.3, JASP-Team, retrieved from https://jasp-stats.org/ on 5 January 2025) and Jamovi (version 2.3.28.0, Jamovi Project 2022, retrieved from https://www.jamovi.org on 5 January 2025) software packages. Data were z-score transformed and tested for a pairwise correlation. All variables with a high shared variance (*r* > 0.80 were further used for the PCA to reduce redundancy and condense correlated variables into orthogonal components. The Kaiser–Meyer–Olkin (KMO) test (>0.5) verified availability of sufficient data (sample adequacy) and Bartlett’s test confirmed sphericity (<0.05) [16,17,18]. Based on the exploratory objective of the present study including a highly specific cohort group of top-elite swimmers, a lower KMO threshold is considered acceptable to reduce dimensionality compared to other analyses aiming at confirmatory factor extraction [16,17,18]. An orthogonal Varimax factor rotation was used to extract the components based on an Eigenvalue > 1.0. Variables with factor loadings > 0.6 were considered for further analyses [7,11,19,20]. The variables of each component with the highest loading were used as independent variables for the multiple linear regression analysis, along with the variables with low co-variance. This approach is commonly applied in sport science to generate practically interpretable results from complex data sets [7,20,21,22]. Normal distribution was confirmed, as the data showed a diagonal straight line in the Q-Q plot (observed vs. theoretical quartiles) and a random pattern in the scatter plot (residuals vs. predicted values). The 15 m start time was used as the dependent variable. All predictors that reached statistical significance were designated as KPIs. The 5 m, 7.5 m and 10 m split times were excluded from the PCA and regression analyses because they are components of the main performance outcome (15 m start time) and not independent predictors. Instead, they are reported descriptively to support the interpretation of start performance analyses. The present study sample includes top-elite swimmers with an age range of 13 to 32 years. Although age- and sex-specific physiological differences are recognized in the general population [23], the present sample of national team members represents a very specific and homogenous group of individuals. Previous research in elite swimming has reported no substantial differences in start and age groups, and variations in KPIs were primarily associated with differences in overall start performance (15 m time) [7,24]. Moreover, due to the large set of kinetic and kinematic variables and reference values across four swimming strokes, splitting the sample by sex and/or age would have substantially increased the complexity of the analysis. Hence, pooling the data allows for a robust and comprehensive analysis of top-elite swimming start performance, while maintaining statistical power and facilitating practical application for coaches and practitioners. Given the complex and multidimensional nature of swimming start performance, no a priori variable selection was applied in order to avoid subjective preselection and ensure that potentially relevant performance indicators were not excluded. Instead, all kinetic and kinematic variables provided by the instrumented starting block were included to use the complete potential of PCA as a data-driven and objective dimension reduction technique. An alpha-level ≤ 0.05 defined statistical significance. The 3rd, 10th, 25th, 50th, 75th, 90th and 97th percentiles of the original non-z-score transformed data are present as reference values.

## 3. Results

Table 2 shows the principal components based on their z-score normalized variables with corresponding Varimax component loadings across all swimming strokes. The highest loaded variables of each component, as well as variables with low co-variance, were used as predictors for the dependent variables (15 m start time) of the multiple linear regression analysis and presented in Table 3 as KPIs when they reached statistical significance.

**Figure 1:**
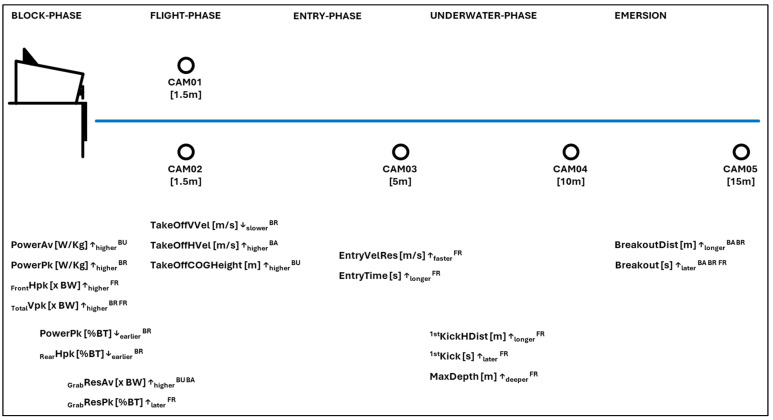
Summary of the key performance indicators (KPIs) revealed by principal component analysis (PCA) and multiple linear regression analysis for elite swimming start performance. The illustration also shows the camera positions of the KiSwim system with their relative distance to the edge of the starting block and the pool wall. Abbreviations: butterfly ^BU^, backstroke ^BA^, breaststroke ^BR^, freestyle ^FR^. Details and explanations for the variables are presented in Table 1.

Figure 1 summarizes the KPIs across all swimming strokes. For practical application and interpretation of performance analysis data, reference values for the swimming stroke-specific KPIs and 5 m, 7.5 m, 10 m and 15 m split times are presented in Table 4. Additionally, percentile-based sex-specific reference values for all variables measured by the KiSwim are presented in the Appendix A (Table A1, Table A2, Table A3, Table A4, Table A5, Table A6, Table A7 and Table A8). Given that the variables most strongly associated with faster start performance primarily reflect differences in 15 m start time rather than sex or age, the data were pooled across sexes for the main analyses. As magnitudes of these variables, however, may differ between males and females, sex-specific reference values are provided in the Appendix A.

Faster swimmers showed higher peak (3rd to 97th percentile: 37.4 to 65.2 [W/kg]) and average power (15.3 to 24.4 [W/kg]) and front horizontal (0.59 to 0.86 [× BW]) and total vertical peak forces (1.03 to 1.72 [× BW]). Both peak power (68 to 89 [%BT]) and rear horizontal forces peaked (49 to 76 [%BT]) earlier during the block phase of faster swimmers. In contrast, the higher resultant grab forces (0.18 to 0.55 [× BW]) of faster swimmers peaked later (26 to 52 [%BT]) during the block phase. At take-off, faster swimmers showed a higher center-of-gravity height (0.75 to 1.26 [m]), higher take-off horizontal velocity (2.84 to 5.03 [m/s]) and lower take-off vertical velocity (−1.61 to −0.18 [m/s]). Moreover, their entry was characterized by a higher resultant entry velocity (5.74 to 6.98 [m/s]) and longer entry time (0.89 to 1.14 [s]). During the underwater phase, faster swimmers covered more distance before the first kick (3.99 to 6.49 [m]) and before breaking through the water surface (10.1 to 15.5 [m]). Breakout distance was also the most influential predictor in the regression model for BA (Std. *β* = 1.18, *t* = 11.1, *p* < 0.01) and BR (Std. *β* = 1.29, *t* = 15.1, *p* < 0.01), while distance before the first kick had the greatest relative contribution in the model for FR (Std. *β* = 1.53, *t* = 11.8, *p* < 0.01). Relative contribution was equally distributed between the three predictors for BU (refer to Table 3). Also, their underwater phase was characterized by a larger maximal swimming depth (−0.73 to −1.33 [m]).

## 4. Discussion

The aims of the present study were to (1) determine kinetic and kinematic KPIs for faster swimming start performances across all four swimming strokes and (2) provide percentile-based reference values for elite junior and adult swimmers (national team members). Co-variance analysis and PCA were used to determine the predictors for the multiple linear regression analysis, which then determined the KPIs contributing to faster start performances. As such, faster swimmers showed higher peak and average power and higher force production that both occurred earlier during their block phase. In contrast, their higher grab forces peaked later. Their take-off was characterized by a higher center-of-gravity height and a higher take-off horizontal velocity and their entry by a higher resultant entry velocity. During their longer underwater phase, faster swimmers had a greater depth and covered more distance before the first kick.

### 4.1. Kinetic Variables

With regard to the kinetic variables, the present study revealed the importance of high on-block power and force production to achieve faster 15 m start times. This is in line with previous studies that also showed great effects of on-block power production [25] and a close relationship between dry-land strength and power (1RM squat strength and jumping height) and swim start performance [26]. Another key finding of the present study is that the superior neuro-muscular abilities, i.e., greater rate of force development, of faster swimmers were associated with an earlier peak of power and rear horizontal force production. Particularly during the early block phase, the smaller knee angle and foot support on the back of the starting block allows the rear leg to produce a higher horizontal impulse, which contributes most to the horizontal take-off velocity and flight distance. The front leg takes over during the later block phase, once the rear leg is no longer in contact with the starting block [12,27,28,29].

Additionally, the front leg directs the force production of the rear leg in the right direction, as the swimmer rotates like an “inverted pendulum” around the front leg from the starting signal to hands-off [28]. Therefore, force and leg stiffness of the front leg are also required to achieve a higher center-of-gravity at take-off, which was associated with faster start performances in the present study. Moreover, faster start trials were associated with a slower take-off vertical velocity, which, based on a downward directed trajectory resulting in negative values (refer to percentiles), affects a steeper take-off angle (refer to percentiles). As steeper take-off angle (more forward and less downward directed angle at take-off) affects the flight trajectory and helps maximize flight distance, it is an important contributor to faster start performances, as shown in a previous study [12].

In the present study, faster starts were associated with higher grab forces that peaked later during the block phase. As the rear leg initiates the force production [25], faster swimmers evidently use the full potential of their upper-body strength with an arm pull that peaks later during the block phase. Higher grab forces during the block start also allow for a better alignment of the center of gravity to the rear leg’s force vector, hence a more effective horizontally directed acceleration. Grab forces and their timings may become even more relevant due to the handgrips on the side of the starting block (refer to the World Aquatics rule book part two: 16.1.8), which supports a more natural and advantageous shoulder position and elbow joint angle for maximal force production [30,31,32]. While grab force also revealed a particular importance during the backstroke start in previous studies [33,34], individual patterns (a higher initial position with the arms pulling the upper body toward the wall vs. a more leaned-back position with more extended arms) require further investigations regarding the optimal sequential contribution of arm and leg forces.

### 4.2. Kinematic Variables

Regarding the kinematic variables, faster starts were associated with higher velocities at water entry, ranging from 5.74 to 6.98 m/s. To take full advantage of this momentum, swimmers must minimize hydrodynamic resistance as they transition to the horizontal phase of the underwater trajectory [35] and maintain a streamlined position until their velocity drops near their maximal undulatory kicking velocity. Initiating the kicks too early increases frontal area and drag forces, which results in premature deceleration. For optimal energy efficiency, the goal during this phase is to minimize deceleration throughout the glide rather than to seek early propulsion through underwater kicks [36]. In the present study, the later initiation of the first undulating kick observed in higher-performing swimmers may reflect one or more of the following mechanisms: (a) higher on-block force production, (b) superior gliding abilities or (c) better timing for the initiation of the first undulating kick [37,38,39,40,41]. While the present study identified on-block force and power production as important KPIs, optimal timing appears particularly crucial to delay the first undulating kick until the initially higher velocity from water entry has decreased to maximal undulating kicking velocity [42,43]. Better gliding and kicking abilities of the fastest swimmers obviously also contribute to the longer underwater distances before breaking through the water surface, an important success factor for swim races [44]. With the longer and deeper underwater trajectory shown in the present study, faster swimmers take full advantage of the lower drag forces below the water surface and flatten the initial curvature of the underwater trajectory after the entry, and hence further minimize drag forces [45,46].

### 4.3. Practical Implications

PCA is a straightforward and widely used method to reduce dimensions and cluster variables with high co-variance, while the highest Varimax factor loading determines the best representation for each principal component [17,20,47]. It should be noted that our pooled analysis across sexes and age groups includes a wide range of physiological characteristics, from young adolescents to fully mature adults. While this introduces some inherent variation in strength and power, it also allows the present study to identify the KPIs that distinguish faster from slower swimming starts within a cohort of top-elite swimmers. While future studies may explore age- or sex-specific adaptations, the pooled approach here allows for a practical and interpretable framework for coaches and performance analysts. The KPIs identified as significant predictors in the regression analysis [7,24] serve as a practical starting point to interpret kinetic and kinematic start performance data. Nevertheless, all variables recorded by the KiSwim system provide valuable information. When the identified KPIs do not fully address a specific performance-related question, additional variables may be considered to achieve a comprehensive analysis of the observed issue. The reference values for the KPIs in Table 4 and for all other variables in Appendix A Table A1, Table A2, Table A3, Table A4, Table A5, Table A6, Table A7 and Table A8 help identify which aspects of an individual swimmer’s start performance are already well-developed and which offer the greatest potential for improvement. The percentile data further support the creation of benchmarks and a stepwise development plan that target the variables with the highest potential to improve an individual’s start performance.

## 5. Conclusions

The present study provides KPIs for swimming start performances of elite junior and adult swimmers. With regard to the kinetic variables, PCA and multiple regression analysis identified peak and average power, front horizontal and total vertical peak forces, timing of peak power and rear horizontal forces, resultant grab forces and their timing as KPIs of faster swimming start performance. With regard to kinematic variables, the present study emphasized the relevance of center-of-gravity height at take-off, take-off horizontal and vertical velocity, resultant entry velocity, entry time, distance before the first kick, maximal swimming depth and distance before breaking through the water surface. The results suggest that swimmers should maximize power and force production early and grab forces later during the block phase. Rear leg, arm pulling and front leg force production should be optimally coordinated to allow for a high center-of-gravity and horizontal velocity at take-off. A faster entry velocity, deeper maximal swimming depth, later initiation of the first kick and longer underwater distance all contribute to faster start performances. The KPIs should serve as a starting point to interpret kinetic and kinematic start performance analysis data, while the other variables should be used to investigate specific details of individual start trials. The percentiles provide reference ranges and peak values, facilitate comparison across KPIs and highlight those that are already well-developed versus those with the greatest potential for improvement.

## Figures and Tables

**Table 1:** List of variables with explanations.

Nr.	Variable	Explanation
1	Time^1st^Move [s]	Reaction time: from starting signal to first movement [seconds]
2	^1st^MoveRear [s]	Time from starting signal to first movement of rear leg [seconds]
3	^1st^MoveGrab [s]	Time from starting signal to first arm movement [seconds]
4	^1st^MoveFront [s]	Time from starting signal to first movement of front leg [seconds]
5	HandsOff [s]	Time from starting signal to hands off block [seconds]
6	HandsOff [%BT]	Hands off block [percentage of block time]
7	ToeOffRear [%BT]	Toe-off of rear leg [percentage of block time]
8	TimeOnBlock [s]	Block time: from starting signal to toe-off of front leg [seconds]
9	TimeOnwall [s]	Backstroke block time: from starting signal to toe-off [seconds]
10	PowerPk [W/kg]	Peak power [watts per kg body weight]
11	PowerPk [%BT]	Peak power [percentage of block time]
12	PowerAv [W/kg]	Average power [watts per kg body weight]
13	_Front_Hpk [× BW]	Front leg horizontal peak force [times body weight]
14	_Front_Hpk [%BT]	Front leg horizontal peak force [percentage of block time]
15	_Front_Vpk [× BW]	Front leg vertical peak force [times body weight]
16	_Front_ResPk [× BW]	Front leg resultant peak force [times body weight]
17	_Front_ResAv [× BW]	Front leg resultant average force [times body weight]
18	_Rear_Hpk [× BW]	Rear leg horizontal peak force [times body weight]
19	_Rear_Hpk [%BT]	Rear leg horizontal peak force [percentage of block time]
20	_Rear_Vpk [× BW]	Rear leg vertical peak force [times body weight]
21	_Rear_Vpk [%BT]	Rear leg vertical peak force [percentage of block time]
22	_Rear_ResPk [× BW]	Rear leg resultant peak force [times body weight]
23	_Rear_ResPk [%BT]	Rear leg resultant peak force [percentage of block time]
24	_Rear_ResAv [× BW]	Rear leg resultant average force [times body weight]
25	_Total_Hpk [× BW]	Total horizontal peak force [times body weight]
26	_Total_Hpk [%BT]	Total horizontal peak force [percentage of block time]
27	_Total_Vpk [× BW]	Total vertical peak force [times body weight]
28	_Total_Vpk [%BT]	Total vertical peak force [percentage of block time]
29	_Total_ResPk [× BW]	Total resultant peak force [times body weight]
30	_Total_ResPk [%BT]	Total resultant peak force [percentage of block time]
31	_Total_ResAv [× BW]	Total resultant average force [times body weight]
32	_Grab_Hpk [× BW]	Grab horizontal peak force [times body weight]
33	_Grab_Hpk [%BT]	Grab horizontal peak force [percentage of block time]
34	_Grab_Vpk [× BW]	Grab vertical peak force [times body weight]
35	_Grab_Vpk [%BT]	Grab vertical peak force [percentage of block time]
36	_Grab_ResPk [× BW]	Grab resultant peak force [times body weight]
37	_Grab_ResPk [%BT]	Grab resultant peak force [percentage of block time]
38	_Grab_ResAv [× BW]	Grab resultant average force [times body weight]
39	Work/kg [Joule/kg]	Total work on block [joule per kg body weight]
40	TakeOffHVel [m/s]	Horizontal take-off velocity [meter per seconds]
41	TakeOffVVel [m/s]	Vertical take-off velocity [meter per seconds]
42	TakeOffResVel [m/s]	Resultant take-off velocity [meter per seconds]
43	TakeOffAngle [°]	Body angle at take-off (center of head to edge of starting block) [degree]
44	TakeOffCOGDist [m]	Center of gravity: distance from edge of starting block at take-off [meter]
45	TakeOffCOGHeight [m]	Center of gravity: height from water surface at take-off [meter]
46	GunCOGDist [m]	Center of gravity: distance from pool wall at starting signal (backstroke only) [meter]
47	GunCOGHeight [m]	Center of gravity: height from water surface at starting signal (backstroke only) [meter]
48	EntryTime [s]	Time from starting signal to top of head at water surface [seconds]
49	EntryDistance [m]	Distance from pool wall to top of head at water surface [meters]
50	SizeEntryHole [m]	Size of entry hole on water surface [meters]
51	EntryVelRes [m/s]	Resultant entry velocity [meter per seconds]
52	EntryAngle [°]	Angle of the center of gravity’s flight trajectory relative to the water surface [degree]
53	^1st^KickHDist [m]	Horizontal distance from pool wall to center of head at first underwater kick [meters]
54	^1st^KickDepth [m]	Vertical distance of head to water surface at first underwater kick [meters]
55	^1st^Kick [s]	Time from starting signal to first underwater kick [seconds]
56	MaxDepthHDist [m]	Horizontal distance from pool wall to center of head at MaxDepth [meters]
57	MaxDepth [m]	Vertical distance of head to water surface at maximal swimming depth [meters]
58	MaxDepth [s]	Time from starting signal to MaxDepth [seconds]
59	Breakout [s]	Time from starting signal to top of head breaking through water surface [meters]
60	BreakoutDist [m]	Distance from pool wall to top of head breaking through water surface [meters]
61	Split5 [s]	Split time: from starting signal to top of head at 5 m [seconds]
62	Split7.5 [s]	Split time: from starting signal to top of head at 7.5 m [seconds]
63	Split10 [s]	Split time: from starting signal to top of head at 10 m [seconds]
64	Split15 [s]	Split time: from starting signal to top of head at 15 m [seconds]

**Table 2:** Principal components (PCs) with z-score normalized variables and corresponding Varimax component loadings including Bartlett’s test of sphericity and Kaiser–Meyer–Olkin (KMO) measure for sampling adequacy.

Butterfly			Backstroke			Breaststroke			Freestyle		
PC	Variable	Loading	PC	Variable	Loading	PC	Variable	Loading	PC	Variable	Loading
**1**	**_Total_Vpk [**× **BW]**	**0.93**	**1**	**_Total_** **Vpk [× BW]**	**0.83**	**1**	**TakeOffHVel [m/s]**	**0.93**	**1**	**_Rear_** **ResPk [× BW]**	**0.94**
	_Total_ResPk [× BW]	0.88		TakeOffAngle [°]	0.83		TakeOffResVel [m/s]	0.91		_Rear_Hpk [× BW]	0.90
	PowerPk [W/kg]	0.81		_Total_ResAv [× BW]	0.82		Work/kg [Joules/kg]	0.80		_Rear_Vpk [× BW]	0.87
**2**	**TakeOffResVel [m/s]**	**0.93**		_Total_Rpk [× BW]	0.79		_Rear_Hpk [× BW]	0.63		_Total_ResAv [× BW]	0.87
	TakeOffHVel [m/s]	0.88		Work/kg [Joule/kg]	0.70	**2**	**_Front_** **ResPk [× BW]**	**0.92**		_Total_ResPk [× BW]	0.85
	Work/kg [Joule/kg]	0.64	**2**	**EntryVelRes [m/s]**	**0.97**		_Front_Vpk [× BW]	0.90		_Rear_ResAv [× BW]	0.71
**3**	**MaxDepth [m]**	**−0.84**		TakeOffResVel [m/s]	0.97		_Grab_ResAv [× BW]	0.82		Work/kg [Joule/kg]	0.70
	MaxDepth [s]	0.83		PowerPk [W/kg]	0.76		_Grab_ResPk [× BW]	0.70		TakeOffHVel [m/s]	0.67
	BreakoutDist [m]	0.74				**3**	**MaxDepth [s]**	**0.92**	**2**	**_Grab_** **ResAv [× BW]**	**0.89**
**4**	**_Front_** **ResPk [× BW]**	**0.85**					MaxDepthHDist [m]	0.92		_Front_ResPk [× BW]	0.89
	_Grab_ResPk [× BW]	0.77					^1st^Kick [s]	0.66		_Grab_ResPk [× BW]	0.86
**5**	**TakeOffCOGDist [m]**	**0.81**				**4**	**_Grab_** **ResPk [%BT]**	**0.83**	**3**	**MaxDepth [m]**	**−0.87**
	^1st^Kick [s]	0.77					_Total_ResAv [× BW]	0.65		BreakoutDist [m]	0.83
										MaxDepth [s]	0.76
									**4**	**TakeOffResVel [m/s]**	**0.81**
									**5**	**^1st^** **Kick [s]**	**0.76**
										EntryDistance [m]	0.72
									**6**	**_Rear_** **Hpk [%BT]**	**−0.89**
Bartlett’s test: <0.01 KMO: 0.76 Total variance explained: 79.7% Variable reduction: 64.3%			Bartlett’s test: <0.01 KMO: 0.67 Total variance explained: 81.6% Variable reduction: 77.8%			Bartlett’s test: <0.01 KMO: 0.66 Total variance explained: 80.9% Variable reduction: 71.4%			Bartlett’s test: <0.01 KMO: 0.76 Total variance explained: 82.0% Variable reduction: 68.4%		

**Table 3:** Multiple linear regression analysis with 15 m start time as the dependent variable and highest loaded variables of each component and variables with low co-variance as predictors. All predictors that reached statistical significance were designated as key performance indicators (KPIs) and are presented as absolute values in the table.

*R* Square	*F* Value	*p* Value		Std. *β*	*t* Value	*p* Value
**Butterfly**						
0.86	12 _[43|85]_	<0.01	PowerAv [W/kg]	0.28	2.00	0.05
			_Grab_ResAv [× BW]	0.38	2.00	0.05
			TakeOffCOGHeight [m]	0.35	2.18	0.03
**Backstroke**						
0.99	104 _[36|34]_	<0.01	_Grab_ResAv [× BW]	0.09	2.17	0.04
			TakeOffHVel [m/s]	0.42	2.36	0.02
			Breakout [s]	1.16	11.0	<0.01
			BreakoutDist [m]	1.18	11.1	<0.01
**Breaststroke**						
0.98	95 _[41|72]_	<0.01	PowerPk [W/kg]	0.32	2.48	0.02
			PowerPk [%BT]	0.07	2.30	0.02
			_Rear_Hpk [%BT]	0.15	3.82	<0.01
			_Total_Vpk [× BW]	0.20	2.58	0.01
			TakeOffVVel [m/s]	0.20	2.50	0.02
			Breakout [s]	1.19	19.1	<0.01
			BreakoutDist [m]	1.29	15.1	<0.01
**Freestyle**						
0.91	40 _[39|158]_	<0.01	_Front_Hpk [× BW]	0.07	2.03	0.04
			_Total_Vpk [× BW]	0.19	3.61	<0.01
			_Grab_ResPk [%BT]	0.10	2.05	0.04
			EntryTime [s]	0.20	3.61	<0.01
			EntryVelRes [m/s]	0.20	2.71	0.01
			^1st^KickHDist [m]	1.53	11.8	<0.01
			^1st^Kick [s]	1.17	11.1	<0.01
			MaxDepth [m]	0.30	2.45	0.02
			Breakout [s]	0.19	5.00	<0.01

**Table 4:** Percentile-based reference values for the key performance indicators (KPIs) alongside the 5 m, 7.5 m, 10 m and 15 m split times. Percentile-based sex-specific reference values for all variables provided by the KiSwim are provided in the Appendix A.

	Percentiles						
	3rd	10th	25th	50th	75th	90th	97th
**Butterfly**							
PowerAv [W/kg]	15.3	15.9	17.2	18.9	20.9	23.1	24.4
_Grab_ResAv [× BW]	0.18	0.21	0.26	0.36	0.42	0.50	0.55
TakeOffCOGHeight [m]	0.75	0.82	0.89	1.01	1.14	1.23	1.26
Split5 [s]	1.79	1.73	1.66	1.58	1.49	1.43	1.42
Split7.5 [s]	3.18	3.07	2.90	2.70	2.50	2.33	2.30
Split10 [s]	4.90	4.67	4.43	4.06	3.84	3.50	3.42
Split15 [s]	8.14	7.77	7.51	6.96	6.60	6.14	5.90
**Backstroke**							
_Grab_ResAv [× BW]	0.29	0.32	0.37	0.41	0.46	0.50	0.53
TakeOffHVel [m/s]	2.84	2.95	3.18	3.54	3.93	4.32	5.03
Breakout [s]	5.08	5.42	6.20	6.83	7.44	7.79	8.07
BreakoutDist [m]	10.1	11.3	12.5	13.5	14.6	14.9	15.5
Split5 [s]	2.23	2.12	2.01	1.88	1.70	1.53	1.50
Split7.5 [s]	3.80	3.68	3.51	3.31	3.06	2.64	2.56
Split10 [s]	5.40	5.30	5.09	4.80	4.46	3.88	3.77
Split15 [s]	8.85	8.54	8.30	7.84	7.38	6.50	6.35
**Breaststroke**							
PowerPk [%BT]	89.0	80.7	77.0	75.0	73.0	71.0	68.0
PowerPk [W/kg]	37.4	40.3	46.0	50.0	57.0	60.0	65.2
_Rear_Hpk [%BT]	75.6	73.0	70.9	64.0	57.3	51.3	49.4
_Total_Vpk [× BW]	1.04	1.09	1.17	1.27	1.42	1.50	1.57
TakeOffVVel [m/s]	−1.61	−1.35	−1.15	−0.88	−0.56	−0.43	−0.18
Breakout [s]	5.16	5.50	5.77	6.17	6.66	6.96	7.28
BreakoutDist [m]	10.6	10.9	11.5	12.2	12.8	13.5	14.0
Split5 [s]	1.80	1.73	1.67	1.56	1.52	1.47	1.43
Split7.5 [s]	3.17	3.12	2.97	2.77	2.58	2.50	2.42
Split10 [s]	5.08	4.79	4.54	4.27	4.01	3.84	3.77
Split15 [s]	9.30	9.08	8.74	8.21	7.79	7.43	7.31
**Freestyle**							
_Front_Hpk [× BW]	0.59	0.61	0.66	0.72	0.77	0.82	0.86
_Total_Vpk [× BW]	1.03	1.06	1.13	1.27	1.40	1.53	1.72
_Grab_ResPk [%BT]	26.0	33.0	36.0	40.0	45.0	50.0	52.0
EntryTime [s]	0.89	0.95	0.98	1.01	1.05	1.09	1.14
EntryVelRes [m/s]	5.74	5.87	6.10	6.24	6.49	6.77	6.98
^1st^KickHDist [m]	3.99	4.34	4.64	5.14	5.48	5.80	6.49
^1st^Kick [s]	1.36	1.44	1.54	1.66	1.76	1.86	1.98
MaxDepth [m]	−0.73	−0.79	−0.87	−1.00	−1.13	−1.23	−1.33
Breakout [s]	3.73	4.00	4.40	5.15	5.79	6.35	6.94
Split5 [s]	1.80	1.77	1.68	1.58	1.50	1.45	1.42
Split7.5 [s]	3.23	3.07	2.94	2.73	2.54	2.42	2.34
Split10 [s]	4.90	4.66	4.46	4.18	3.88	3.67	3.55
Split15 [s]	8.15	7.77	7.49	7.04	6.55	6.30	6.08

## Data Availability

The original contributions and variable-level data presented in this study are included in the article. Further inquiries can be directed to the corresponding author.

## Abbreviations

The following abbreviations are used in this manuscript:

BU	Butterfly
BA	Backstroke
BR	Breaststroke
FR	Freestyle
KPIs	Key performance indicators
PCA	Principal component analysis

## Appendix A

**Table A1:** Percentile-based reference values for male butterfly swimmers across all variables provided by the KiSwim.

	Percentile						
	3rd	10th	25th	50th	75th	90th	97th
**Butterfly**							
Time^1st^Move [s]	0.29	0.26	0.22	0.19	0.15	0.13	0.12
^1st^MoveRear [s]	0.22	0.2	0.17	0.16	0.14	0.13	0.09
^1st^MoveGrab [s]	0.22	0.19	0.18	0.15	0.13	0.12	0.06
^1st^MoveFront [s]	0.27	0.24	0.21	0.19	0.17	0.15	0.13
HandsOff [%BT]	47.4	58.0	64.0	68.0	70.0	73.1	75.0
ToeOffRear [%BT]	78.1	80.0	81.0	83.0	84.0	85.0	86.0
TimeOnBlock [s]	0.78	0.76	0.71	0.68	0.66	0.63	0.62
PowerAv [W/kg]	16.0	17.3	18.5	20.0	22.7	23.9	24.5
PowerPk [%BT]	78.0	77.0	75.0	74.0	72.0	71.0	69.0
PowerPk [W/kg]	40.1	44.9	50.0	56.5	62.0	66.1	68.9
_Front_Hpk [%BT]	92.0	91.1	91.0	90.0	89.0	88.0	88.0
_Front_Hpk [× BW]	0.55	0.60	0.66	0.71	0.74	0.78	0.83
_Front_Vpk [× BW]	1.09	1.16	1.24	1.35	1.43	1.52	1.64
_Front_ResPk [× BW]	1.07	1.16	1.24	1.38	1.43	1.53	1.66
_Front_ResAv [× BW]	0.77	0.81	0.86	0.92	1.00	1.05	1.09
_Rear_Hpk [%BT]	73.0	70.1	69.0	65.0	59.0	55.9	53.1
_Rear_Hpk [× BW]	0.70	0.76	0.80	0.91	1.02	1.09	1.20
_Rear_Vpk [%BT]	60.9	58.1	55.0	51.5	47.0	41.9	36.1
_Rear_Vpk [× BW]	0.72	0.79	0.86	0.96	1.06	1.16	1.25
_Rear_ResPk [%BT]	66.9	64.1	60.0	56.5	53.0	49.9	42.1
_Rear_ResPk [× BW]	0.96	1.06	1.15	1.25	1.45	1.57	1.69
_Rear_ResAv [× BW]	0.50	0.51	0.54	0.59	0.65	0.69	0.72
_Total_ResAv [× BW]	1.05	1.09	1.14	1.18	1.22	1.26	1.28
_Total_Hpk [%BT]	75.9	75.0	68.8	62.0	57.0	51.9	49.1
_Total_Hpk [× BW]	1.03	1.09	1.15	1.29	1.41	1.53	1.61
_Total_Vpk [%BT]	77.0	75.0	74.0	72.0	70.0	65.0	35.7
_Total_Vpk [× BW]	1.09	1.18	1.29	1.39	1.52	1.65	1.76
_Total_ResPk [%BT]	76.0	75.0	73.0	71.0	67.0	60.0	57.1
_Total_ResPk [× BW]	1.51	1.55	1.72	1.87	2.01	2.13	2.19
_Grab_ResAv [× BW]	0.21	0.26	0.34	0.39	0.47	0.53	0.57
_Grab_ResPk [%BT]	30.2	35.0	38.0	40.5	43.0	46.0	51.7
_Grab_ResPk [× BW]	0.58	0.72	0.81	1.01	1.11	1.17	1.25
Work/kg [Joule/kg]	11.5	12.2	13.1	13.9	15.3	15.7	15.9
TakeOffHVel [m/s]	4.00	4.09	4.22	4.35	4.50	4.63	4.82
TakeOffVVel [m/s]	−1.55	−1.35	−1.00	−0.70	−0.53	−0.35	−0.19
TakeOffResVel [m/s]	4.07	4.16	4.26	4.42	4.59	4.71	4.86
TakeOffAngle [°]	5.0	8.9	11.25	14.0	18.0	21.0	22.0
TakeOffCOGDist [m]	0.81	0.83	0.92	1.13	1.32	1.54	1.60
TakeOffCOGHeight [m]	0.82	0.85	0.93	1.06	1.20	1.25	1.26
EntryTime [s]	0.92	0.96	0.98	1.02	1.06	1.09	1.14
EntryDistance [m]	2.52	2.67	2.83	3.07	3.20	3.44	3.73
SizeEntryHole [m]	1.10	0.85	0.72	0.64	0.55	0.48	0.37
EntryVelRes [m/s]	5.84	6.11	6.24	6.4	6.63	6.75	6.81
EntryAngle [°]	51.0	50.0	49.0	47.0	45.0	43.9	43.0
^1st^KickHDist [m]	4.29	4.48	4.95	5.46	5.77	6.04	6.36
^1st^KickDepth [m]	−0.75	−0.84	−0.95	−1.07	−1.17	−1.29	−1.35
^1st^Kick [s]	1.44	1.49	1.56	1.64	1.74	1.88	2.07
MaxDepthHDist [m]	6.95	6.46	6.19	5.68	5.34	4.94	4.66
MaxDepth [m]	−0.77	−0.94	−1.05	−1.12	−1.25	−1.37	−1.44
MaxDepth [s]	2.21	2.00	1.89	1.77	1.65	1.58	1.50
Breakout [s]	4.21	4.68	5.13	5.53	5.77	6.31	6.84
BreakoutDist [m]	10.0	10.8	11.8	12.8	14.0	14.5	14.8
Split5 [s]	1.68	1.63	1.58	1.51	1.45	1.42	1.38
Split7.5 [s]	2.92	2.79	2.69	2.53	2.38	2.30	2.28
Split10 [s]	4.51	4.34	4.07	3.88	3.56	3.46	3.37
Split15 [s]	7.62	7.37	6.96	6.65	6.27	5.95	5.80

**Table A2:** Percentile-based reference values for male backstroke swimmers across all variables provided by the KiSwim.

	Percentile						
	3rd	10th	25th	50th	75th	90th	97th
**Backstroke**							
Time^1st^Move [s]	0.33	0.26	0.22	0.18	0.14	0.13	0.07
^1st^MoveGrab [s]	0.26	0.23	0.18	0.17	0.15	0.13	0.12
HandsOff [s]	0.50	0.48	0.44	0.42	0.39	0.35	0.34
TimeOnWall [s]	0.74	0.72	0.70	0.68	0.63	0.60	0.57
PowerAv [W/kg]	11.2	12.0	13.1	16.1	22.1	29.6	30.8
PowerPk [%BT]	89.7	89.0	89.0	88.0	87.0	85.8	85.0
PowerPk [W/kg]	38.3	40.4	46.0	54.0	67.0	83.6	86.0
_Total_Hpk [%BT]	88.0	87.6	87.0	86.0	45.0	40.4	39.0
_Total_Hpk [× BW]	1.14	1.23	1.32	1.51	1.61	1.67	1.88
_Total_Vpk [%BT]	82.0	63.8	51.0	47.0	43.0	40.0	35.3
_Total_Vpk [× BW]	0.88	0.95	1.05	1.12	2.06	2.40	2.76
_Total_ResPk [%BT]	86.7	86.0	85.0	48.0	44.0	39.4	39.0
_Total_ResPk [× BW]	1.49	1.53	1.71	1.83	2.42	2.98	3.16
_Total_ResAv [× BW]	1.08	1.16	1.24	1.33	1.60	1.90	2.03
_Grab_Vpk [%BT]	32.0	34.0	36.0	39.0	42.0	44.6	47.4
_Grab_Vpk [× BW]	0.62	0.56	0.51	0.44	0.39	0.33	0.28
_Grab_Hpk [%BT]	4.6	7.0	21.0	29.0	32.0	35.2	39.0
_Grab_Hpk [× BW]	0.76	0.79	0.86	0.94	1.05	1.22	1.33
_Grab_Respk [%BT]	6.0	15.2	23.0	30.0	33.0	37.2	39.0
_Grab_Respk [× BW]	0.78	0.83	0.93	0.99	1.15	1.27	1.41
_Grab_ResAv [× BW]	0.32	0.36	0.39	0.42	0.48	0.51	0.53
Work/kg [Joule/kg]	7.4	7.8	8.5	10.5	16.1	19.6	21.2
TakeOffHVel [m/s]	3.05	3.25	3.39	3.82	4.05	4.45	5.10
TakeOffVVel [m/s]	−0.75	−0.71	−0.48	−0.17	0.35	0.82	1.15
TakeOffResVel [m/s]	3.16	3.27	3.47	3.82	4.23	4.49	5.12
TakeOffAngle [°]	−12.7	−9.6	−7.0	−2.0	5.0	11.6	14.7
GunCOGHeight [m]	0.12	0.16	0.22	0.27	0.31	0.35	0.41
GunCOGDist [m]	0.23	0.27	0.33	0.48	0.62	0.85	0.97
TakeOffCOGDist [m]	1.10	1.18	1.37	1.51	1.73	1.85	1.95
TakeOffCOGHeight [m]	0.16	0.21	0.29	0.46	0.57	0.74	0.81
EntryTime [s]	0.71	0.77	0.81	0.87	0.92	0.95	0.95
EntryDistance [m]	2.12	2.23	2.42	2.61	2.92	3.04	3.49
SizeEntryHole [m]	1.49	1.11	0.94	0.78	0.55	0.33	0.23
EntryVelRes [m/s]	3.16	3.27	3.47	3.76	4.11	4.49	5.12
EntryAngle [°]	50.7	47.0	43.0	38.0	35.0	34.0	31.3
^1st^KickHDist [m]	3.41	3.91	4.09	4.32	4.61	4.99	5.33
^1st^KickDepth [m]	−0.51	−0.70	−0.83	−1.01	−1.13	−1.25	−1.38
^1st^Kick [s]	1.24	1.31	1.36	1.50	1.63	1.69	1.76
MaxDepthHDist [m]	6.91	6.61	6.35	6.10	5.52	5.30	4.90
MaxDepth [m]	−0.94	−1.02	−1.10	−1.24	−1.31	−1.46	−1.51
MaxDepth [s]	2.94	2.75	2.53	2.26	2.03	1.93	1.83
Breakout [s]	5.09	5.39	5.88	6.66	7.27	7.76	7.98
BreakoutDist [m]	10.8	11.7	12.8	13.7	14.6	14.9	15.2
Split5 [s]	2.01	1.97	1.89	1.81	1.62	1.51	1.47
Split7.5 [s]	3.54	3.42	3.33	3.15	2.81	2.60	2.56
Split10 [s]	5.16	4.94	4.81	4.60	4.10	3.83	3.77
Split15 [s]	8.31	8.10	7.92	7.65	6.76	6.47	6.34

**Table A3:** Percentile-based reference values for male breaststroke swimmers across all variables provided by the KiSwim.

	Percentile						
	3rd	10th	25th	50th	75th	90th	97th
**Breaststroke**							
Time^1st^Move [s]	0.28	0.26	0.22	0.19	0.16	0.14	0.13
^1st^MoveRear [s]	0.23	0.22	0.20	0.17	0.16	0.15	0.14
^1st^MoveGrab [s]	0.22	0.20	0.18	0.17	0.15	0.13	0.13
^1st^MoveFront [s]	0.29	0.25	0.21	0.19	0.17	0.16	0.15
HandsOff [%BT]	49.2	52.0	55.0	60.0	66.8	74.0	76.0
ToeOffRear [%BT]	79.0	79.0	80.3	83.0	85.0	86.9	87.0
TimeOnBlock [s]	0.78	0.76	0.74	0.69	0.67	0.65	0.63
PowerAv [W/kg]	16.7	17.7	18.8	19.6	21.6	22.6	23.8
PowerPk [%BT]	88.2	79.0	77.0	75.0	73.0	69.1	67.8
PowerPk [W/kg]	42.5	46.0	49.0	56.0	59.8	62.9	66.0
_Front_Hpk [%BT]	91.0	91.0	92.0	90.0	90.0	88.0	88.0
_Front_Hpk [× BW]	0.55	0.57	0.70	0.75	0.78	0.81	0.84
_Front_Vpk [× BW]	1.04	1.16	1.23	1.40	1.53	1.67	1.84
_Front_ResPk [× BW]	1.07	1.19	1.28	1.40	1.53	1.71	1.84
_Front_ResAv [× BW]	0.76	0.82	0.89	0.94	1.01	1.05	1.10
_Rear_Hpk [%BT]	75.2	72.0	66.0	59.0	53.3	50.0	45.8
_Rear_Hpk [× BW]	0.73	0.79	0.84	0.92	1.05	1.14	1.15
_Rear_Vpk [%BT]	64.3	59.0	54.0	50.0	47.0	44.0	42.7
_Rear_Vpk [× BW]	0.69	0.73	0.82	0.91	1.05	1.20	1.26
_Rear_ResPk [%BT]	72.3	66.4	57.0	54.0	49.0	46.0	42.8
_Rear_ResPk [× BW]	1.05	1.09	1.17	1.23	1.45	1.64	1.68
_Rear_ResAv [× BW]	0.48	0.50	0.53	0.57	0.60	0.63	0.65
_Total_ResAv [× BW]	1.09	1.11	1.14	1.17	1.20	1.24	1.26
_Total_Hpk [%BT]	78.0	77.0	75.0	60.0	51.3	45.1	41.8
_Total_Hpk [× BW]	1.06	1.09	1.14	1.28	1.44	1.62	1.66
_Total_Vpk [%BT]	78.0	77.0	75.0	73.0	70.0	65.1	60.0
_Total_Vpk [× BW]	1.10	1.17	1.23	1.33	1.47	1.54	1.67
_Total_ResPk [%BT]	78.0	77.0	76.0	72.0	66.0	62.0	59.8
_Total_ResPk [× BW]	1.43	1.60	1.67	1.76	2.00	2.11	2.22
_Grab_ResAv [× BW]	0.15	0.23	0.31	0.37	0.42	0.49	0.54
_Grab_ResPk [%BT]	27.2	32.1	35.0	39.0	41.0	44.0	50.0
_Grab_ResPk [× BW]	0.51	0.73	0.80	0.91	1.10	1.15	1.25
Work/kg [Joule/kg]	11.8	12.5	13.0	14.1	14.9	15.6	15.8
TakeOffHVel [m/s]	4.06	4.11	4.16	4.36	4.49	4.65	4.71
TakeOffVVel [m/s]	−1.52	−1.22	−1.00	−0.73	−0.50	−0.41	−0.17
TakeOffResVel [m/s]	4.06	4.13	4.25	4.40	4.60	4.76	4.94
TakeOffAngle [°]	5.8	8.0	11.0	13.0	17.0	19.9	23.0
TakeOffCOGDist [m]	0.87	0.91	0.96	1.05	1.29	1.47	1.52
TakeOffCOGHeight [m]	0.88	0.94	1.00	1.10	1.19	1.25	1.29
EntryTime [s]	0.94	0.96	1.00	1.02	1.06	1.09	1.10
EntryDistance [m]	2.48	2.69	2.86	2.94	3.22	3.50	3.54
SizeEntryHole [m]	0.94	0.86	0.79	0.65	0.53	0.43	0.35
EntryVelRes [m/s]	6.03	6.16	6.33	6.51	6.66	6.74	6.91
EntryAngle [°]	51.0	50.0	49.0	47.5	46.0	45.0	43.8
^1st^KickHDist [m]	6.36	6.65	7.14	7.69	8.07	8.26	8.89
^1st^KickDepth [m]	−0.66	−0.72	−0.82	−0.98	−1.08	−1.17	−1.21
^1st^Kick [s]	2.08	2.26	2.52	2.72	2.99	3.17	3.38
MaxDepthHDist [m]	7.39	7.10	6.66	6.16	5.85	5.53	5.16
MaxDepth [m]	−0.87	−0.94	−1.07	−1.21	−1.28	−1.34	−1.42
MaxDepth [s]	2.55	2.40	2.20	1.92	1.80	1.69	1.64
Breakout [s]	5.06	5.49	5.70	6.13	6.65	7.11	7.52
BreakoutDist [m]	10.8	11.4	12.0	12.5	13.4	13.8	14.1
Split5 [s]	1.63	1.56	1.55	1.52	1.49	1.44	1.43
Split7.5 [s]	2.89	2.82	2.69	2.59	2.51	2.45	2.39
Split10 [s]	4.67	4.28	4.22	4.02	3.90	3.81	3.76
Split15 [s]	8.48	8.26	8.12	7.81	7.48	7.33	7.25

**Table A4:** Percentile-based reference values for male freestyle swimmers across all variables provided by the KiSwim.

	Percentile						
	3rd	10th	25th	50th	75th	90th	97th
**Freestyle**							
Time^1st^Move [s]	0.32	0.26	0.23	0.20	0.16	0.14	0.11
^1st^MoveRear [s]	0.23	0.21	0.20	0.18	0.17	0.15	0.14
^1st^MoveGrab [s]	0.25	0.23	0.19	0.17	0.15	0.14	0.12
^1st^MoveFront [s]	0.27	0.24	0.22	0.19	0.17	0.15	0.14
HandsOff [%BT]	48.4	56.0	63.0	71.0	74.0	75.0	76.3
ToeOffRear [%BT]	77.7	79.0	81.0	83.0	84.0	85.0	86.0
TimeOnBlock [s]	0.78	0.76	0.72	0.70	0.67	0.64	0.63
PowerAv [W/kg]	15.5	17.8	19.0	20.9	22.6	23.6	24.9
PowerPk [%BT]	91.0	87.0	77.0	74.0	73.0	70.0	68.0
PowerPk [W/kg]	41.7	46.9	50.3	56.0	61.0	66.0	69.3
_Front_Hpk [%BT]	92.0	92.0	91.0	90.0	89.0	88.0	86.7
_Front_Hpk [× BW]	0.60	0.64	0.70	0.75	0.80	0.83	0.86
_Front_Vpk [× BW]	0.99	1.05	1.11	1.23	1.38	1.53	1.72
_Front_ResPk [× BW]	1.05	1.11	1.18	1.29	1.45	1.55	1.72
_Front_ResAv [× BW]	0.67	0.73	0.81	0.88	0.95	1.02	1.17
_Rear_Hpk [%BT]	73.0	71.1	67.0	63.0	58.0	54.0	49.0
_Rear_Hpk [× BW]	0.75	0.79	0.90	1.01	1.16	1.25	1.27
_Rear_Vpk [%BT]	62.0	59.0	57.0	53.0	49.0	44.9	39.7
_Rear_Vpk [× BW]	0.77	0.86	0.93	1.09	1.22	1.28	1.36
_Rear_ResPk [%BT]	70.0	64.1	61.0	57.0	54.0	49.0	44.0
_Rear_ResPk [× BW]	1.09	1.17	1.26	1.46	1.61	1.69	1.84
_Rear_ResAv [× BW]	0.47	0.50	0.56	0.61	0.64	0.67	0.73
_Total_ResAv [× BW]	1.07	1.11	1.14	1.19	1.21	1.26	1.28
_Total_Hpk [%BT]	76.3	73.0	65.8	62.0	58.0	54.0	44.0
_Total_Hpk [× BW]	1.01	1.08	1.20	1.40	1.59	1.71	1.81
_Total_Vpk [%BT]	81.3	76.0	74.0	72.0	69.3	65.0	61.3
_Total_Vpk [× BW]	1.06	1.12	1.22	1.34	1.46	1.56	1.75
_Total_ResPk [%BT]	77.0	76.0	73.0	70.0	63.0	59.0	56.0
_Total_ResPk [× BW]	1.43	1.53	1.70	1.90	2.05	2.15	2.33
_Grab_ResAv [× BW]	0.08	0.22	0.31	0.37	0.44	0.53	0.59
_Grab_ResPk [%BT]	34.7	35.0	39.0	42.5	47.8	51.0	53.0
_Grab_ResPk [× BW]	0.27	0.63	0.78	0.93	1.05	1.18	1.21
Work/kg [Joule/kg]	12.2	12.6	13.4	14.4	15.3	16.2	16.6
TakeOffHVel [m/s]	4.02	4.15	4.33	4.45	4.64	4.79	4.86
TakeOffVVel [m/s]	−1.53	−1.32	−1.07	−0.84	−0.66	−0.38	−0.21
TakeOffResVel [m/s]	4.07	4.26	4.40	4.54	4.70	4.93	4.98
TakeOffAngle [°]	2.7	5.0	10.0	12.0	14.8	18.0	21.3
TakeOffCOGDist [m]	0.87	0.91	0.97	1.13	1.31	1.45	1.62
TakeOffCOGHeight [m]	0.80	0.87	0.94	1.01	1.18	1.25	1.30
EntryTime [s]	0.91	0.95	1.00	1.01	1.06	1.09	1.15
EntryDistance [m]	2.47	2.65	2.81	2.98	3.19	3.51	3.63
SizeEntryHole [m]	1.03	0.92	0.77	0.69	0.56	0.41	0.34
EntryVelRes [m/s]	5.96	6.17	6.21	6.44	6.72	6.90	7.06
EntryAngle [°]	51.0	50.0	48.0	47.0	45.0	43.9	41.0
^1st^KickHDist [m]	4.41	4.64	5.10	5.42	5.77	6.06	6.68
^1st^KickDepth [m]	−0.70	−0.78	−0.88	−0.98	−1.10	−1.20	−1.28
^1st^Kick [s]	1.36	1.46	1.57	1.67	1.79	1.90	2.05
MaxDepthHDist [m]	6.53	6.23	5.84	5.25	4.84	4.68	4.51
MaxDepth [m]	−0.75	−0.81	−0.93	−1.04	−1.17	−1.26	−1.36
MaxDepth [s]	2.03	1.93	1.74	1.63	1.48	1.44	1.41
Breakout [s]	3.61	3.91	4.31	5.00	5.67	5.91	6.40
BreakoutDist [m]	9.2	9.6	10.5	11.9	13.2	14.3	14.9
Split5 [s]	1.73	1.57	1.53	1.50	1.45	1.43	1.41
Split7.5 [s]	2.94	2.73	2.63	2.54	2.44	2.38	2.30
Split10 [s]	4.44	4.21	4.04	3.86	3.69	3.57	3.46
Split15 [s]	7.39	6.95	6.72	6.49	6.32	6.15	5.95

**Table A5:** Percentile-based reference values for female butterfly swimmers across all variables provided by the KiSwim.

	Percentile						
	3rd	10th	25th	50th	75th	90th	97th
**Butterfly**							
Time^1st^Move [s]	0.34	0.29	0.24	0.20	0.17	0.16	0.15
^1st^MoveRear [s]	0.21	0.20	0.18	0.16	0.15	0.14	0.12
^1st^MoveGrab [s]	0.21	0.19	0.18	0.16	0.15	0.13	0.11
^1st^MoveFront [s]	0.25	0.23	0.21	0.18	0.16	0.14	0.12
HandsOff [%BT]	49.0	52.0	56.0	63.0	68.0	70.0	72.0
ToeOffRear [%BT]	79.0	79.0	80.0	82.0	83.0	84.2	85.0
TimeOnBlock [s]	0.75	0.73	0.72	0.70	0.67	0.64	0.61
PowerAv [W/kg]	14.9	15.5	16.1	17.3	18.6	20.0	21.0
PowerPk [%BT]	90.0	89.2	76.0	74.0	72.0	70.0	69.0
PowerPk [W/kg]	36.5	39.0	42.0	45.0	48.5	52.0	54.0
_Front_Hpk [%BT]	92.0	92.0	91.0	90.0	89.0	88.0	88.0
_Front_Hpk [× BW]	0.59	0.62	0.64	0.70	0.76	0.78	0.81
_Front_Vpk [× BW]	1.06	1.09	1.18	1.35	1.43	1.57	1.75
_Front_ResPk [× BW]	1.06	1.10	1.18	1.39	1.45	1.60	1.75
_Front_ResAv [× BW]	0.65	0.73	0.77	0.83	0.93	0.99	1.05
_Rear_Hpk [%BT]	70.0	69.0	68.0	62.0	58.0	53.0	47.0
_Rear_Hpk [× BW]	0.70	0.73	0.82	0.88	0.97	1.02	1.07
_Rear_Vpk [%BT]	66.0	57.2	53.0	50.0	44.5	37.0	33.7
_Rear_Vpk [× BW]	0.63	0.69	0.77	0.88	1.00	1.11	1.22
_Rear_ResPk [%BT]	69.0	68.0	63.5	57.0	52.0	48.0	41.0
_Rear_ResPk [× BW]	0.96	1.00	1.09	1.23	1.35	1.41	1.56
_Rear_ResAv [× BW]	0.46	0.48	0.51	0.54	0.60	0.64	0.70
_Total_ResAv [× BW]	1.01	1.03	1.06	1.11	1.16	1.19	1.28
_Total_Hpk [%BT]	75.0	74.0	70.0	62.0	58.0	51.0	48.5
_Total_Hpk [× BW]	0.96	1.02	1.09	1.18	1.27	1.35	1.51
_Total_Vpk [%BT]	75.3	75.0	73.8	71.0	65.0	59.7	57.0
_Total_Vpk [× BW]	1.01	1.04	1.07	1.16	1.29	1.45	1.61
_Total_ResPk [%BT]	75.0	74.2	73.0	70.0	61.0	58.0	56.4
_Total_ResPk [× BW]	1.36	1.46	1.52	1.61	1.77	1.85	1.93
_Grab_ResAv [× BW]	0.18	0.20	0.24	0.27	0.36	0.42	0.51
_Grab_ResPk [%BT]	29.7	31.0	33.0	39.0	41.0	45.0	49.0
_Grab_ResPk [× BW]	0.42	0.46	0.60	0.73	0.90	1.01	1.12
Work/kg [Joule/kg]	10.2	10.8	11.6	12.1	12.7	13.2	13.8
TakeOffHVel [m/s]	3.63	3.79	3.93	4.03	4.18	4.27	4.33
TakeOffVVel [m/s]	−2.01	−1.79	−1.37	−1.15	−0.69	−0.32	0.18
TakeOffResVel [m/s]	3.71	3.86	4.03	4.20	4.42	4.51	4.71
TakeOffAngle [°]	0.2	3.0	6.0	10.0	12.0	19.4	22.3
TakeOffCOGDist [m]	0.77	0.81	0.93	1.12	1.26	1.39	1.51
TakeOffCOGHeight [m]	0.75	0.79	0.87	0.98	1.08	1.15	1.21
EntryTime [s]	0.82	0.89	0.94	0.99	1.03	1.07	1.11
EntryDistance [m]	2.12	2.29	2.47	2.70	2.89	3.13	3.36
SizeEntryHole [m]	0.86	0.77	0.73	0.64	0.53	0.48	0.40
EntryVelRes [m/s]	5.58	5.79	5.93	6.23	6.31	6.44	6.54
EntryAngle [°]	52.3	51.0	50.0	49.0	47.0	45.0	43.7
^1st^KickHDist [m]	3.87	4.36	4.61	4.85	5.30	5.47	5.71
^1st^KickDepth [m]	−0.75	−0.80	−0.87	−0.98	−1.06	−1.14	−1.26
^1st^Kick [s]	1.36	1.45	1.56	1.68	1.79	1.82	1.88
MaxDepthHDist [m]	6.48	6.03	5.44	4.84	4.38	4.08	3.95
MaxDepth [m]	−0.76	−0.84	−0.91	−1.04	−1.14	−1.24	−1.33
MaxDepth [s]	2.29	2.02	1.85	1.63	1.52	1.43	1.36
Breakout [s]	3.67	4.10	4.77	5.82	6.48	6.81	6.93
BreakoutDist [m]	8.4	9.1	10.1	12.5	13.4	14.5	14.7
Split5 [s]	1.81	1.77	1.72	1.65	1.60	1.56	1.51
Split7.5 [s]	3.28	3.16	3.04	2.89	2.74	2.65	2.58
Split10 [s]	4.98	4.77	4.62	4.37	4.11	3.97	3.92
Split15 [s]	8.28	8.07	7.71	7.47	7.10	6.88	6.77

**Table A6:** Percentile-based reference values for female backstroke swimmers across all variables provided by the KiSwim.

	Percentile						
	3rd	10th	25th	50th	75th	90th	97th
**Backstroke**							
Time^1st^Move [s]	0.29	0.25	0.20	0.18	0.15	0.13	0.12
^1st^MoveGrab [s]	0.27	0.25	0.23	0.18	0.13	0.13	0.11
HandsOff [s]	0.54	0.48	0.45	0.40	0.36	0.33	0.31
TimeOnWall [s]	0.77	0.69	0.67	0.65	0.61	0.59	0.56
PowerAv [W/kg]	7.8	9.8	10.5	11.6	13.6	15.2	17.2
PowerPk [%BT]	90.0	89.5	89.0	88.5	87.2	86.0	84.8
PowerPk [W/kg]	26.3	30.0	33.5	40.5	47.8	53.5	57.5
_Total_Hpk [%BT]	89.3	88.5	88.0	87.0	84.5	46.5	34.3
_Total_Hpk [× BW]	0.89	1.02	1.15	1.26	1.43	1.55	1.64
_Total_Vpk [%BT]	84.3	78.5	76.0	49.0	44.0	36.0	28.5
_Total_Vpk [× BW]	0.84	0.90	0.93	1.03	1.11	1.20	1.49
_Total_ResPk [%BT]	87.3	87.0	86.8	84.5	44.3	38.5	34.3
_Total_ResPk [× BW]	1.24	1.29	1.43	1.59	1.76	1.88	2.05
_Total_ResAv [× BW]	0.94	1.02	1.07	1.14	1.24	1.35	1.43
_Grab_Vpk [%BT]	30.5	32.5	34.0	37.0	40.8	42.0	44.0
_Grab_Vpk [× BW]	0.64	0.60	0.51	0.40	0.34	0.28	0.25
_Grab_Hpk [%BT]	9.5	10.5	12.5	21.0	24.5	29.5	33.8
_Grab_Hpk [× BW]	0.60	0.62	0.66	0.82	0.90	1.06	1.08
_Grab_Respk [%BT]	9.5	10.5	15.3	23.0	30.0	33.0	37.5
_Grab_Respk [× BW]	0.70	0.72	0.76	0.83	1.03	1.09	1.09
_Grab_ResAv [× BW]	0.26	0.30	0.34	0.39	0.44	0.46	0.48
Work/kg [Joule/kg]	5.5	6.0	6.5	7.3	8.3	10.2	11.6
TakeOffHVel [m/s]	2.64	2.84	2.96	3.15	3.41	3.86	4.14
TakeOffVVel [m/s]	−0.94	−0.73	−0.58	−0.40	−0.07	0.08	0.22
TakeOffResVel [m/s]	2.67	2.87	2.99	3.16	3.46	3.90	4.25
TakeOffAngle [°]	−14.8	−11.5	−11.0	−8.0	−1.3	1.5	3.8
GunCOGHeight [m]	0.17	0.19	0.22	0.27	0.33	0.40	0.45
GunCOGDist [m]	0.16	0.20	0.25	0.45	0.59	0.68	0.83
TakeOffCOGDist [m]	0.91	0.97	1.32	1.46	1.60	1.73	1.90
TakeOffCOGHeight [m]	0.11	0.14	0.21	0.38	0.53	0.74	0.78
EntryTime [s]	0.72	0.75	0.78	0.81	0.84	0.91	0.97
EntryDistance [m]	2.05	2.09	2.18	2.34	2.54	2.65	2.75
SizeEntryHole [m]	2.08	1.58	1.10	0.83	0.58	0.39	0.16
EntryVelRes [m/s]	2.67	2.87	2.99	3.16	3.46	3.90	4.25
EntryAngle [°]	50.3	48.0	45.8	41.0	35.3	34.5	32.3
^1st^KickHDist [m]	2.87	3.18	3.44	3.90	4.25	4.61	5.19
^1st^KickDepth [m]	−0.45	−0.55	−0.82	−1.04	−1.17	−1.29	−1.57
^1st^Kick [s]	1.14	1.23	1.34	1.47	1.57	1.69	1.84
MaxDepthHDist [m]	6.57	6.22	5.74	4.95	4.61	4.45	3.32
MaxDepth [m]	−0.65	−0.82	−1.09	−1.16	−1.37	−1.51	−1.59
MaxDepth [s]	2.91	2.74	2.47	2.17	1.88	1.60	1.37
Breakout [s]	5.25	5.75	6.51	7.22	7.73	7.81	8.29
BreakoutDist [m]	9.8	10.4	12.0	13.2	14.1	14.7	15.7
Split5 [s]	2.31	2.20	2.12	2.03	1.96	1.86	1.77
Split7.5 [s]	3.90	3.78	3.67	3.57	3.40	3.23	3.15
Split10 [s]	5.58	5.38	5.30	5.15	4.86	4.70	4.58
Split15 [s]	9.18	8.80	8.56	8.43	8.03	7.68	7.59

**Table A7:** Percentile-based reference values for female breaststroke swimmers across all variables provided by the KiSwim.

	Percentile						
	3rd	10th	25th	50th	75th	90th	97th
**Breaststroke**							
Time^1st^Move [s]	0.28	0.27	0.24	0.19	0.16	0.14	0.12
^1st^MoveRear [s]	0.25	0.21	0.18	0.17	0.16	0.14	0.13
^1st^MoveGrab [s]	0.26	0.25	0.19	0.17	0.16	0.14	0.12
^1st^MoveFront [s]	0.30	0.26	0.22	0.20	0.18	0.17	0.14
HandsOff [%BT]	39.0	48.0	52.0	58.0	63.0	68.8	72.5
ToeOffRear [%BT]	79.0	80.1	81.0	83.0	84.3	87.9	88.0
TimeOnBlock [s]	0.80	0.79	0.74	0.72	0.69	0.66	0.65
PowerAv [W/kg]	13.8	15.1	16.0	17.3	18.4	19.1	19.7
PowerPk [%BT]	89.5	81.0	77.0	75.0	73.0	72.0	71.0
PowerPk [W/kg]	36.5	40.0	41.0	47.0	50.5	56.9	58.0
_Front_Hpk [%BT]	94.0	88.0	90.0	92.0	91.0	91.0	91.0
_Front_Hpk [× BW]	0.57	0.59	0.62	0.69	0.75	0.78	0.81
_Front_Vpk [× BW]	1.10	1.19	1.25	1.32	1.42	1.57	1.61
_Front_ResPk [× BW]	1.10	1.19	1.25	1.32	1.42	1.57	1.61
_Front_ResAv [× BW]	0.69	0.76	0.81	0.89	0.95	1.05	1.10
_Rear_Hpk [%BT]	74.9	74.0	71.3	68.0	62.8	59.0	53.5
_Rear_Hpk [× BW]	0.62	0.65	0.75	0.85	0.91	0.94	0.97
_Rear_Vpk [%BT]	68.0	58.9	53.3	46.5	43.8	41.0	40.0
_Rear_Vpk [× BW]	0.57	0.59	0.67	0.73	0.82	0.94	1.01
_Rear_ResPk [%BT]	74.5	71.9	68.3	59.0	50.0	43.0	41.0
_Rear_ResPk [× BW]	0.76	0.83	0.97	1.10	1.17	1.28	1.31
_Rear_ResAv [× BW]	0.41	0.42	0.45	0.51	0.55	0.56	0.58
_Total_ResAv [× BW]	1.01	1.04	1.06	1.12	1.15	1.17	1.19
_Total_Hpk [%BT]	80.0	76.9	75.0	72.0	64.5	57.0	49.8
_Total_Hpk [× BW]	0.89	0.99	1.06	1.13	1.19	1.29	1.38
_Total_Vpk [%BT]	80.5	78.9	75.0	72.5	71.0	70.0	64.1
_Total_Vpk [× BW]	1.03	1.08	1.13	1.19	1.33	1.43	1.54
_Total_ResPk [%BT]	80.0	78.9	74.3	73.0	70.8	65.4	56.1
_Total_ResPk [× BW]	1.41	1.46	1.51	1.65	1.73	1.87	1.90
_Grab_ResAv [× BW]	0.14	0.17	0.26	0.30	0.35	0.42	0.47
_Grab_ResPk [%BT]	24.0	30.1	34.0	39.0	40.3	43.9	49.4
_Grab_ResPk [× BW]	0.39	0.44	0.60	0.78	0.90	0.98	1.05
Work/kg [Joule/kg]	11.0	11.2	11.8	12.4	12.8	13.6	13.9
TakeOffHVel [m/s]	3.76	3.85	3.94	4.11	4.20	4.34	4.40
TakeOffVVel [m/s]	−1.67	−1.36	−1.23	−1.04	−0.74	−0.52	−0.31
TakeOffResVel [m/s]	3.85	3.97	4.10	4.25	4.36	4.46	4.53
TakeOffAngle [°]	1.5	4.1	7.0	10.0	12.0	16.9	18.5
TakeOffCOGDist [m]	0.81	0.87	0.92	1.11	1.33	1.44	1.50
TakeOffCOGHeight [m]	0.69	0.73	0.84	0.96	1.03	1.10	1.17
EntryTime [s]	0.91	0.95	0.98	1.01	1.04	1.10	1.12
EntryDistance [m]	2.37	2.45	2.48	2.59	2.92	3.07	3.16
SizeEntryHole [m]	0.80	0.71	0.64	0.56	0.45	0.35	0.32
EntryVelRes [m/s]	5.64	5.69	5.84	6.10	6.23	6.34	6.45
EntryAngle [°]	51.0	51.0	49.0	48.0	46.0	44.0	42.5
^1st^KickHDist [m]	5.43	5.92	6.24	6.74	7.04	7.52	7.94
^1st^KickDepth [m]	−0.72	−0.78	−0.90	−1.01	−1.11	−1.24	−1.30
^1st^Kick [s]	1.92	2.09	2.43	2.55	2.72	2.96	3.18
MaxDepthHDist [m]	6.65	6.43	6.00	5.68	5.14	4.81	4.69
MaxDepth [m]	−0.88	−0.95	−1.03	−1.13	−1.24	−1.35	−1.44
MaxDepth [s]	2.35	2.30	2.21	1.98	1.80	1.66	1.56
Breakout [s]	5.36	5.66	5.87	6.22	6.65	6.86	7.12
BreakoutDist [m]	10.6	10.6	11.2	11.8	12.3	12.6	12.9
Split5 [s]	1.81	1.78	1.72	1.68	1.64	1.60	1.54
Split7.5 [s]	3.21	3.16	3.11	2.99	2.89	2.78	2.67
Split10 [s]	5.15	5.03	4.78	4.54	4.38	4.28	4.17
Split15 [s]	9.39	9.15	9.07	8.75	8.52	8.20	8.04

**Table A8:** Percentile-based reference values for female freestyle swimmers across all variables provided by the KiSwim.

	Percentile						
	3rd	10th	25th	50th	75th	90th	97th
**Freestyle**							
Time^1st^Move [s]	0.40	0.33	0.27	0.22	0.19	0.16	0.14
^1st^MoveRear [s]	0.23	0.21	0.18	0.17	0.15	0.14	0.10
^1st^MoveGrab [s]	0.29	0.23	0.20	0.17	0.16	0.14	0.13
^1st^MoveFront [s]	0.27	0.25	0.22	0.19	0.17	0.16	0.10
HandsOff [%BT]	42.2	47.7	53.0	60.0	66.0	69.0	72.0
ToeOffRear [%BT]	77.0	79.0	81.0	83.0	84.0	86.0	87.0
TimeOnBlock [s]	0.82	0.78	0.76	0.71	0.68	0.65	0.63
PowerAv [W/kg]	13.8	14.3	15.7	17.2	18.8	20.3	21.1
PowerPk [%BT]	91.0	81.6	77.0	75.5	72.8	70.0	67.0
PowerPk [W/kg]	37.0	39.0	42.0	46.0	50.0	53.3	55.8
_Front_Hpk [%BT]	92.0	92.0	91.0	90.5	90.0	89.0	87.0
_Front_Hpk [× BW]	0.58	0.61	0.65	0.69	0.75	0.77	0.84
_Front_Vpk [× BW]	0.93	1.02	1.16	1.25	1.40	1.48	1.65
_Front_ResPk [× BW]	0.99	1.07	1.18	1.27	1.41	1.49	1.65
_Front_ResAv [× BW]	0.56	0.68	0.75	0.82	0.89	0.97	1.05
_Rear_Hpk [%BT]	74.0	72.0	69.3	65.0	59.0	51.7	50.0
_Rear_Hpk [× BW]	0.63	0.68	0.78	0.83	0.89	1.01	1.07
_Rear_Vpk [%BT]	66.6	58.0	53.3	47.0	44.0	39.0	34.0
_Rear_Vpk [× BW]	0.62	0.65	0.72	0.83	0.94	1.04	1.12
_Rear_ResPk [%BT]	72.8	69.3	65.0	56.5	49.0	46.0	39.0
_Rear_ResPk [× BW]	0.85	0.93	1.04	1.12	1.25	1.43	1.51
_Rear_ResAv [× BW]	0.42	0.45	0.49	0.53	0.58	0.63	0.65
_Total_ResAv [× BW]	1.00	1.03	1.05	1.10	1.16	1.21	1.23
_Total_Hpk [%BT]	79.0	76.0	74.0	66.0	57.8	49.0	47.0
_Total_Hpk [× BW]	0.88	0.97	1.05	1.11	1.22	1.35	1.47
_Total_Vpk [%BT]	78.8	76.0	74.0	72.0	65.0	65.0	63.2
_Total_Vpk [× BW]	1.02	1.05	1.10	1.21	1.34	1.44	1.59
_Total_ResPk [%BT]	78.0	76.0	75.0	73.0	68.0	62.7	52.4
_Total_ResPk [× BW]	1.27	1.41	1.49	1.64	1.75	1.89	2.06
_Grab_ResAv [× BW]	0.07	0.10	0.22	0.29	0.34	0.39	0.44
_Grab_ResPk [%BT]	26.0	30.0	35.0	39.5	43.0	47.0	50.0
_Grab_ResPk [× BW]	0.16	0.32	0.57	0.73	0.86	0.95	1.06
Work/kg [Joule/kg]	10.5	10.8	11.4	12.3	12.9	13.6	14.1
TakeOffHVel [m/s]	3.71	3.79	3.93	4.05	4.24	4.35	4.41
TakeOffVVel [m/s]	−1.86	−1.65	−1.38	−1.06	−0.72	−0.41	−0.15
TakeOffResVel [m/s]	3.79	3.91	4.05	4.22	4.43	4.53	4.65
TakeOffAngle [°]	−0.6	3.0	6.0	10.0	14.0	18.3	21.0
TakeOffCOGDist [m]	0.77	0.83	0.93	1.13	1.32	1.42	1.53
TakeOffCOGHeight [m]	0.70	0.75	0.85	0.99	1.10	1.18	1.28
EntryTime [s]	0.88	0.94	0.97	1.01	1.05	1.08	1.13
EntryDistance [m]	2.22	2.32	2.54	2.80	2.98	3.14	3.28
SizeEntryHole [m]	1.03	0.97	0.84	0.72	0.64	0.49	0.34
EntryVelRes [m/s]	5.70	5.81	5.97	6.19	6.28	6.41	6.61
EntryAngle [°]	53.8	52.0	51.0	48.5	46.0	45.0	44.0
^1st^KickHDist [m]	3.92	4.12	4.47	4.81	5.23	5.38	5.61
^1st^KickDepth [m]	−0.67	−0.73	−0.81	−0.90	−1.02	−1.10	−1.23
^1st^Kick [s]	1.37	1.43	1.54	1.65	1.74	1.85	1.93
MaxDepthHDist [m]	6.01	5.73	5.13	4.83	4.53	4.25	4.04
MaxDepth [m]	−0.71	−0.77	−0.86	−0.95	−1.05	−1.19	−1.31
MaxDepth [s]	2.05	1.94	1.77	1.63	1.51	1.44	1.36
Breakout [s]	3.92	4.05	4.58	5.22	5.86	6.48	7.13
BreakoutDist [m]	8.9	9.3	10.1	11.5	12.5	13.3	13.9
Split5 [s]	1.85	1.79	1.72	1.67	1.60	1.55	1.50
Split7.5 [s]	3.28	3.18	3.02	2.89	2.77	2.69	2.65
Split10 [s]	5.01	4.81	4.57	4.41	4.26	4.06	4.00
Split15 [s]	8.26	7.98	7.65	7.42	7.15	6.98	6.72
